# Intensity-modulated Radiotherapy for Rectal Cancer in the UK in 2020

**DOI:** 10.1016/j.clon.2020.12.011

**Published:** 2021-04

**Authors:** C.R. Hanna, F. Slevin, A. Appelt, M. Beavon, R. Adams, C. Arthur, M. Beasley, A. Duffton, A. Gilbert, S. Gollins, M. Harrison, M.A. Hawkins, K. Laws, S. O'Cathail, P. Porcu, M. Robinson, D. Sebag-Montefiore, M. Teo, S. Teoh, R. Muirhead

**Affiliations:** ∗ CRUK Clinical Trials Unit, University of Glasgow, Glasgow, UK; † Beatson West of Scotland Cancer Centre, Glasgow, UK; ‡ University of Leeds, Leeds, UK; § Leeds Teaching Hospitals NHS Trust, Leeds, UK; ¶ Royal College of Radiologists, London, UK; || Velindre Cancer Centre, Cardiff, UK; ∗∗ The Christie NHS Foundation Trust, Manchester, UK; †† North Wales Cancer Treatment Centre, Glan Clwyd Hospital, Rhyl, UK; ‡‡ Mount Vernon Cancer Centre, Northwood, UK; §§ Medical Physics and Biochemical Engineering, University College London, London, UK; ¶¶ Aberdeen Cancer Centre, Aberdeen Royal Infirmary, Aberdeen, UK; |||| Royal Free London NHS Foundation Trust, London, UK; ∗∗∗ Oxford University Hospitals NHS Foundation Trust, Oxford, UK

**Keywords:** IMRT, intensity-modulated radiotherapy, neoplasm, rectal cancer, survey

## Abstract

**Aims:**

Preoperative (chemo)radiotherapy followed by total mesorectal excision is the current standard of care for patients with locally advanced rectal cancer. The use of intensity-modulated radiotherapy (IMRT) for rectal cancer is increasing in the UK. However, the extent of IMRT implementation and current practice was not previously known. A national survey was commissioned to investigate the landscape of IMRT use for rectal cancer and to inform the development of national rectal cancer IMRT guidance.

**Materials and methods:**

A web-based survey was developed by the National Rectal Cancer IMRT Guidance working group in collaboration with the Royal College of Radiologists and disseminated to all UK radiotherapy centres. The survey enquired about the implementation of IMRT with a focus on the following aspects of the workflow: dose fractionation schedules and use of a boost; pre-treatment preparation and simulation; target volume/organ at risk definition; treatment planning and treatment verification. A descriptive statistical analysis was carried out.

**Results:**

In total, 44 of 63 centres (70%) responded to the survey; 30/44 (68%) and 36/44 (82%) centres currently use IMRT to treat all patients and selected patients with rectal cancer, respectively. There was general agreement concerning several aspects of the IMRT workflow, including patient positioning, use of intravenous contrast and bladder protocols. Greater variation in practice was identified regarding rectal protocols; use of a boost to primary/nodal disease; target volume delineation; organ at risk delineation and dose constraints and treatment verification. Delineation of individual small bowel loops and daily volumetric treatment verification were considered potentially feasible by most centres.

**Conclusion:**

This survey identified that IMRT is already used to treat rectal cancer in many UK radiotherapy centres, but there is heterogeneity between centres in its implementation and practice. These results have been a valuable aid in framing the recommendations within the new National Rectal Cancer IMRT Guidance.

## Highlights

• First national UK survey of rectal cancer IMRT use and practice.
• Considerable variation identified in IMRT implementation and practice.
• Results of survey informed recommendations within new national consensus guideline.

## Introduction

In patients with locally advanced rectal cancer, radiotherapy has been shown to significantly reduce the risk of locoregional recurrence [[1], [2], [3], [4], [5], [6]].

Recently, preoperative (chemo)radiotherapy for rectal cancer has been increasingly delivered using intensity-modulated radiotherapy (IMRT). However, the extent of IMRT use in the UK for rectal cancer has been unclear and no consensus previously existed regarding its implementation. Given these uncertainties, the potential complexities of an IMRT workflow and the lack of a national strategy, a multidisciplinary working group was formed from radiotherapy centres across the UK to develop the National Rectal Cancer IMRT Guidance [7].

In order to inform guidance development and aid the framing of specific recommendations, the working group commissioned a national survey of radiotherapy centres. The objectives of the survey were to describe the current use and delivery of IMRT, to illustrate areas of consensus and heterogeneity in the IMRT pathway and to understand the feasibility of particular recommendations contained within the draft guidance document.

## Materials and Methods

The survey was developed using a web-based platform (Survey Monkey®) by members of the working group and an internal pilot by seven members was carried out to ensure content validity prior to dissemination.

For centres not currently using IMRT, the survey explored the reasons for this and asked respondents whether a national guidance document would aid IMRT implementation. For these respondents, the survey ended after these questions were completed. For those centres currently using IMRT, the survey questions corresponded to sections contained within the proposed national guidance. The dose fractionation questions primarily concerned ‘long course’ radiotherapy (LCRT) but all other sections were applicable to both ‘short course’ radiotherapy (SCRT) and LCRT. These survey sections included: dose fractionation schedules and use of a boost; pre-treatment preparation and simulation; target volume/organ at risk (OAR) definition; treatment planning and treatment verification. Importantly, the survey asked centres how they treated the majority of the patients with rectal cancer managed within a curative intent pathway. For clarity and to aid data analysis, tick box answers were provided for each question where possible, with the use of free text boxes kept to a minimum. A copy of the final survey can be found in the Appendix.

The survey invitation was disseminated via e-mail on 27 February 2020 to the audit leads at all radiotherapy centres in the UK with the instructions that it should be completed by the clinical lead for rectal cancer radiotherapy in collaboration with a medical physicist and therapeutic radiographer. The survey was open for 4 months. A reminder e-mail was sent to non-responding centres after 6 weeks and individual approaches to complete the survey were made by members of the working group.

Descriptive statistics were carried out using Microsoft® Excel 2016. The numbers of centres responding to each answer for each question were expressed as a percentage of the total. The survey was led by two clinical oncology trainees (CRH and FS) supported by senior members of the working group.

## Results

### Current Use of Intensity-modulated Radiotherapy for Rectal Cancer

In total, 70% (44/63) of centres responded to the survey. Most respondents (36/44, 82%) indicated that they use IMRT to treat selected patients with rectal cancer and 68% (30/44) indicated that they use IMRT to treat all patients with rectal cancer. Volumetric modulated arc therapy was the most common method of IMRT delivery (31/36, 86%).

### Dose Fractionation Schedules

The survey specifically addressed LCRT dose fractionation schedules. For those centres currently using IMRT to treat rectal cancer patients, the most commonly used schedule for the elective lymph node irradiation during preoperative LCRT was 45 Gy in 25 fractions (31/36, 86%). Some centres indicated that they use more than one dose fractionation schedule. Ten centres (28%) use 50.4 Gy in 28 fractions and seven centres (19%) use 50 Gy in 25 fractions. Nevertheless, all 12 centres who indicated that they routinely use more than one elective dose fractionation schedule highlighted that 45 Gy in 25 fractions was their most commonly used schedule.

**Fig 1:**
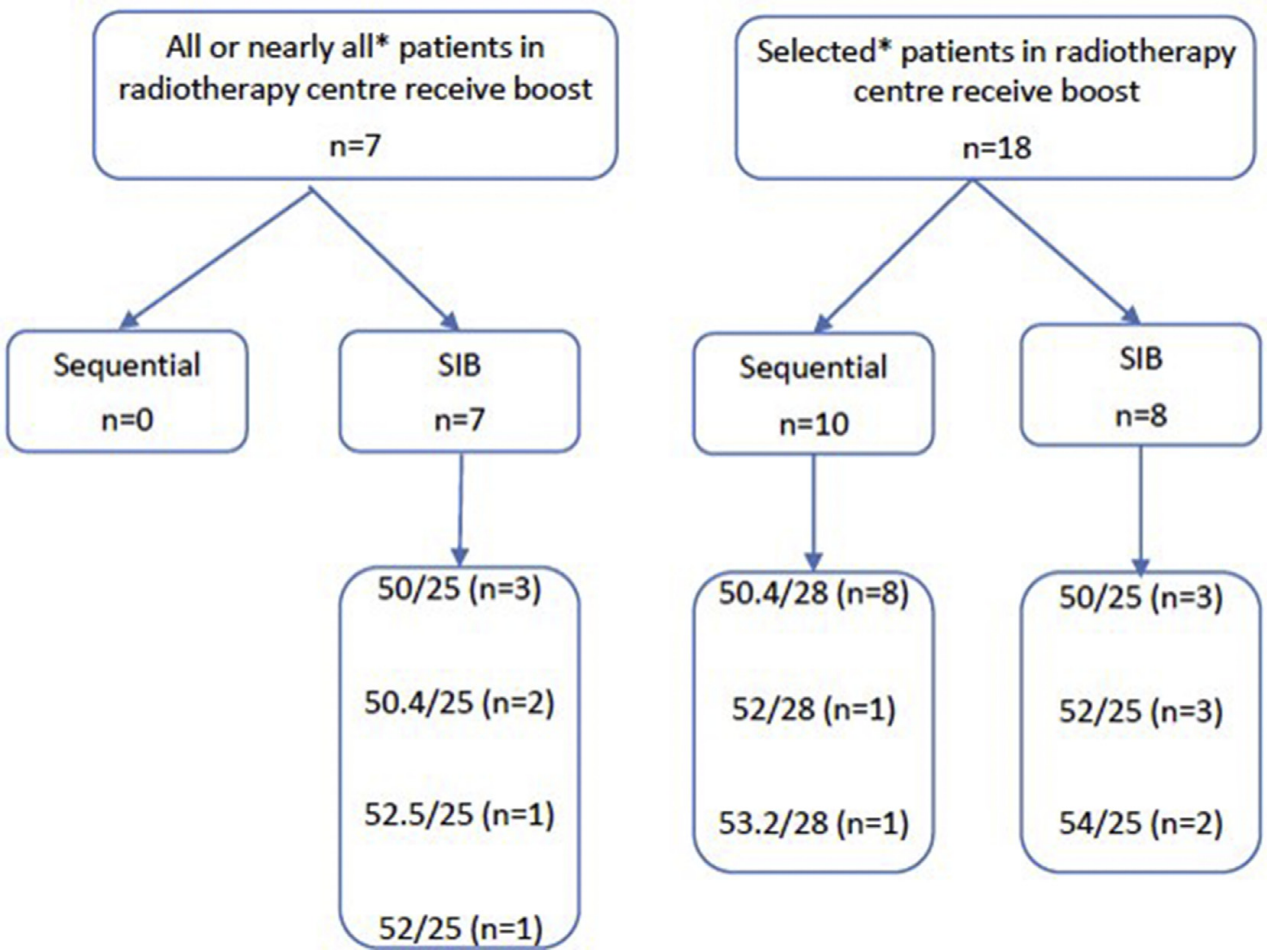
Boost technique and dose fractionation schedules used by UK radiotherapy centres currently using intensity-modulated radiotherapy (n = 25). ‘All or nearly all’ and ‘selected’ were the definitions used in the survey. Interpretation of these definitions was left to the discretion of the responding centre. SIB, simultaneous integrated boost.

In total, 25 centres (25/36, 69%) routinely deliver a boost in selected patients, with seven centres (7/36, 19%) doing this for all or nearly all patients. The dose fractionation schedules used to deliver boost treatments are shown in Figure 1 and indicate use of both sequential and simultaneous integrated boost (SIB) delivery. In those centres that reported use of a boost for all or nearly all patients, this is carried out using a SIB in all cases. In contrast, concerning centres in which a boost is delivered only to selected patients, both sequential and SIB delivery was reported. None of the centres reported use of a boost in excess of 54 Gy.

### Pre-treatment Preparation and Simulation

Of centres that currently use IMRT, most (33/36, 92%) plan and treat patients in the supine position. Most centres (81%) administer intravenous contrast with the simulation computed tomography scan for all patients and six (17%) centres use intravenous contrast only for selected patients. One centre (3%) indicated that they do not use intravenous contrast at all. Small bowel contrast is used by only 14 centres (39%).

In total, 34 of 36 centres (94%) use a bladder protocol for the simulation computed tomography scan. Overall, 27 centres (27/36, 75%) indicated that they use a protocol of 250–600 ml of water 20–40 min before the scan/treatment, one centre (1/36, 3%) indicated that they carry out a planning scan with an empty bladder and six centres (6/36, 17%) use a protocol that is distinct from either of these approaches. Overall, 26 centres (26/36, 72%) indicated that they verify bladder filling during treatment. Specifically, 14 centres (14/36, 39%) use cone beam computed tomography (CBCT) image verification, five centres (5/36, 14%) use ultrasound and four centres (4/36, 11%) indicated that they use a mixture of CBCT and ultrasound.

Overall, 21 of 36 centres (58%) currently using IMRT indicated that they have a rectal protocol for simulation (in four centres asking patients to open bowels prior to simulation; in 16 taking action based on the computed tomography scout view; and in one specifically using a micro-enema if rectal diameter was over 4 cm on computed tomography simulation). Twelve centres (12/36, 33%) reported that they attempt to maintain a rectal protocol during treatment, with nine of these centres (9/36, 25%) specifically reporting the use of CBCT monitoring followed by intervention if rectal filling is problematic.

### Target Volume Definition

**Fig 2:**
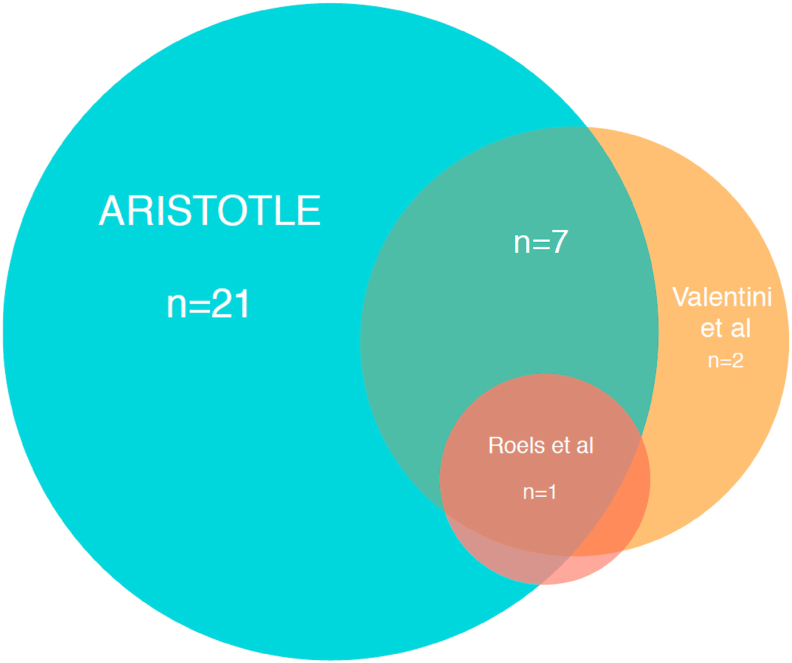
Sources used to guide radiotherapy target volume definition. Thirty-one centres use a specific clinical trial protocol/guideline (*n* = 21 ARISTOTLE alone [8]; *n* = 2 Valentini *et al.* [9] alone; *n* = 7 ARISTOTLE [8] and Valentini *et al.* [9]; *n* = 1 ARISTOTLE [9], Valentini *et al.* [9] and Roels *et al.* [10]).

**Fig 3:**
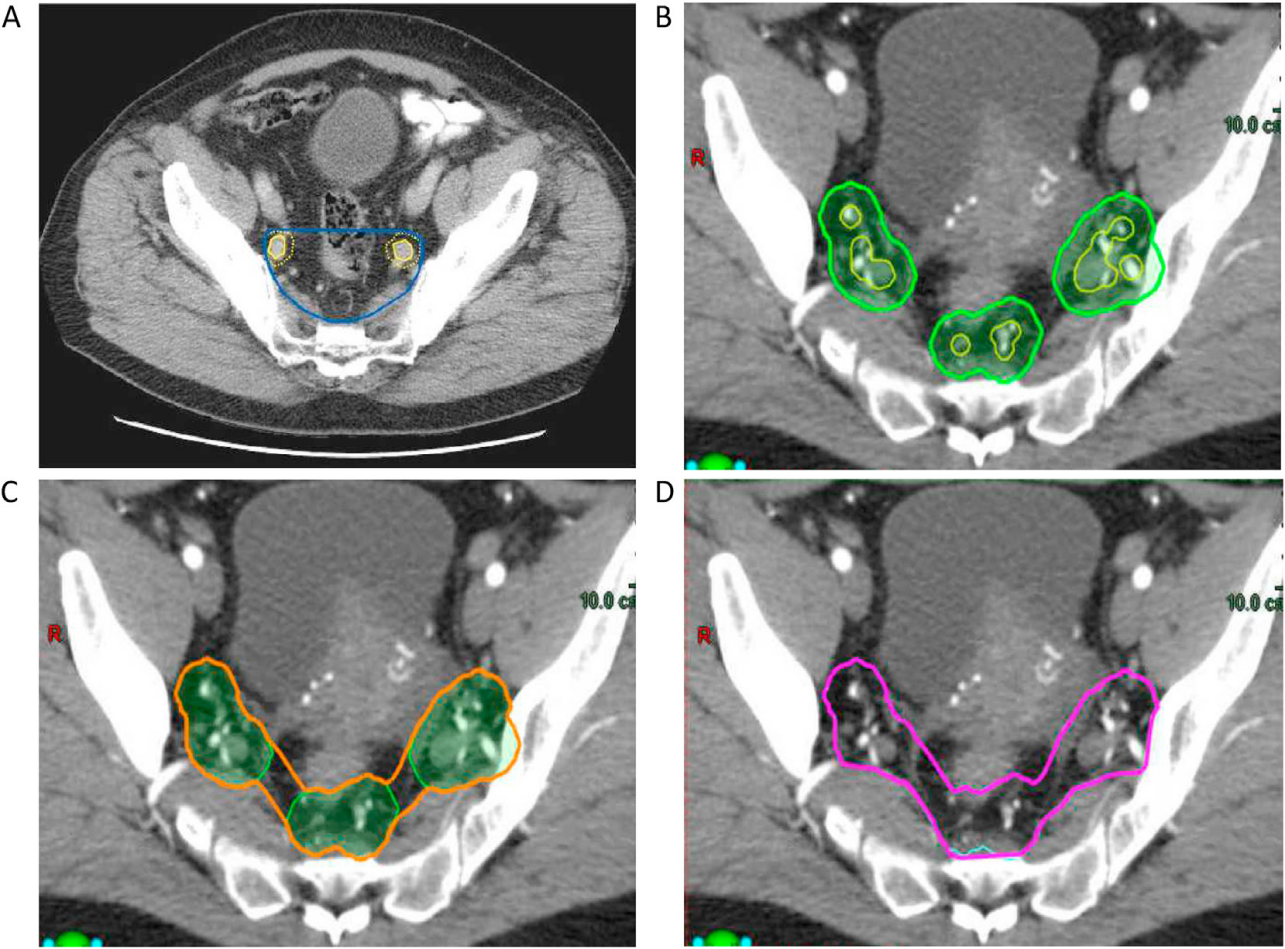
Planning computed tomography images at a similar axial level in the pelvis showing the elective clinical target volume delineated as per (A) the ARISTOTLE trial protocol [8] and (B–D) the new guidance [7]. In (A), a 7 mm margin around the internal iliac vessels determines the borders for the elective clinical target volume, which has a flat anterior border, at this level. In the new guidance, at this level a highly conformal volume is created by the following steps illustrated in (B–D). (B) A 7 mm margin in all directions (except in the superior–inferior direction) is applied to the internal iliac vessels, inferior mesenteric artery and superior rectal vessels at this level. (C) A 10 mm rollerball is used to join these volumes together. (D) The volume is edited off muscle and bone.

All 36 centres currently using IMRT indicated that they use a protocol to guide target volume definition, with 31 centres (31/36, 86%) referencing a specific clinical trial protocol/guideline. Figure 2 illustrates the referenced trial protocols/guidelines and indicates that 31 centres (31/36, 86%) use the ARISTOTLE clinical trial protocol [8]. Table S1 summarises the major differences in target volume delineation between ARISTOTLE and the new guidance and Figure 3 illustrates the differences in the elective clinical target volume between ARISTOTLE and the new guidance. In total, 18 centres (18/36, 50%) stated that they have developed their own local target volume protocol by adapting a previously published protocol (in eight centres based on ARISTOTLE [8]; in four based on ARISTOTLE [8] and Valentini *et al.* [9]; in two based on Valentini *et al.* [9] alone; in one based on ARISTOTLE [8], Valentini *et al.* [9] and Roels *et al.* [10]; in three trial not specified). One centre indicated they had developed their own local protocol without adapting any previous guideline and one centre indicated that their local protocol was developed using the rectal cancer IMRT protocol from a different UK radiotherapy centre. Overall, only 39% (14/36) of centres currently using IMRT indicated that they have routine peer review of rectal cancer target volume contours.

All 36 centres currently using IMRT indicated that magnetic resonance imaging (MRI) is employed in some capacity to aid target volume and OAR delineation. Specifically, 23 centres (23/36, 64%) use the diagnostic MRI side by side with simulation computed tomography, ten (10/36, 28%) use a co-registered diagnostic MRI scan and one (1/36, 3%) uses a radiotherapy-specific MRI scan co-registered with the planning computed tomography scan. One centre (1/36, 3%) indicated that a radiotherapy-specific MRI was carried out if requested by the treating consultant and one centre (1/36, 3%) indicated that a diagnostic MRI was co-registered only if requested by the treating consultant. No centres currently use an MRI-only planning pathway.

### Organ at Risk Definition

**Fig 4:**
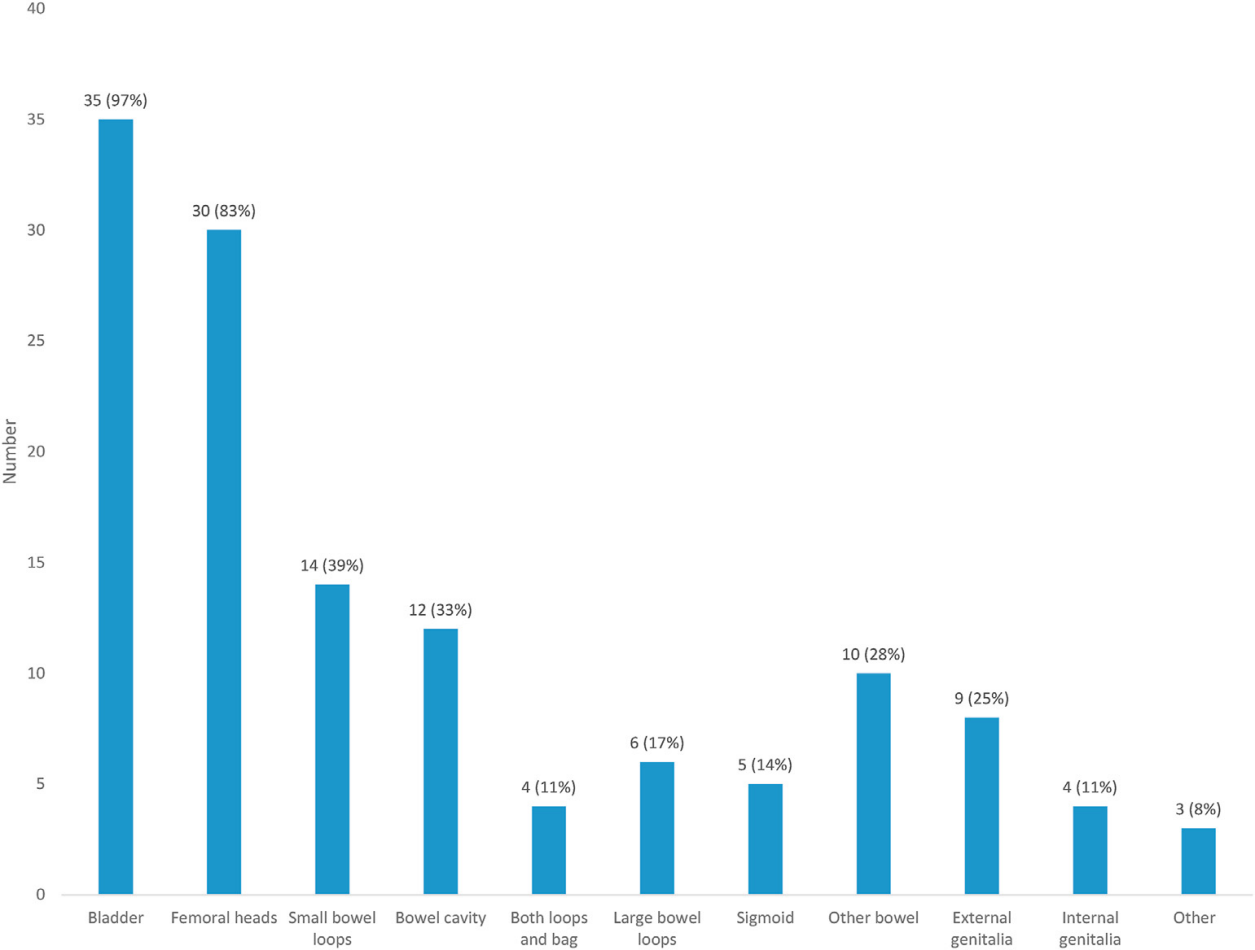
Organs at risk outlined routinely in centres currently using intensity-modulated radiotherapy (n = 36). The number (y-axis) corresponds to the number of centres in which a particular structure is outlined and adds up to greater than 100% as multiple structures are outlined at each centre. Three centres reported delineation of ‘other’ structures, which included stoma (where present) and bone.

Figure 4 illustrates the OARs routinely delineated in centres currently using IMRT. Fourteen centres (14/36, 39%) outline individual small bowel loops and 12 centres (12/36, 33%) outline a ‘bowel space’/peritoneal cavity structure. Four centres (4/36, 11%) indicated that they outline both of these structures. Ten centres (10/36, 28%) indicated that they delineate another type of bowel structure and these variations included individual small and large bowel loops as a single structure and the external contour of groups of bowel loops but including the space between loops (‘bowel bag’ as per EMBRACE II clinical trial) [11]. Two centres (2/36, 6%) reported that they do not routinely contour any bowel structure.

For the purposes of treatment planning and/or plan evaluation, 31 centres (31/36, 86%) currently using IMRT utilise OAR dose constraints. The remaining five centres (5/36, 14%) indicated that although they do not use constraints, they aim to reduce dose to OAR as much as is feasible. Of those 31 centres that do use OAR constraints, 18 (18/31, 58%) indicated that these constraints were derived from a clinical trial protocol (ARISTOTLE [8], nine centres; RTOG 0822 [12], three centres; PLATO [13], three centres; trial not specified, three centres). Other reported sources for OAR constraints included the National Anal Cancer IMRT Guidance [14], Quantec guidelines [15], Valentini *et al.* [9], historical data from that centre, and unspecified gynaecology/genitourinary cancer radiotherapy guidelines.

### Image-guided Radiotherapy and Treatment Verification

Table 1 tabulates the on-treatment imaging modalities used by the 36 centres currently using IMRT.

**Table 1:** Frequency of use for various on-treatment imaging technologies and frequency of imaging and whether centres changed imaging frequency when they adopted intensity-modulated radiotherapy (IMRT) for the treatment of rectal cancer. The total number exceeds the 36 centres currently using IMRT as in some centres multiple imaging modalities are used

	Number (*n* = 36)	Percentage (rounded)†
Modality		
2D kV alone	1	3%
CBCT alone	20	56%
CBCT and 2D kV	11	31%
CBCT and 2D kV/MV mix	2	6%
CBCT and on-board imaging	1	3%
CBCT and MRI	1	3%
Frequency of imaging (and if there was a change in protocol when centres switched from 3D conformal treatment to IMRT)		
Daily	19	53%
*Change*	*13 (13/19,68%)*	
*No change*	*6 (6/19,32%)*	
‘No action limit’ protocol∗	16	44%
*Change*	*12 (12/16,75%)*	
*No change*	*4 (4/16,25%)*	
First three to five fractions only	1	0%
*Change*	*1 (1/1.100%)*	
*No change*	*0 (0/1,0%)*	

2D, two-dimensional; 3D, three-dimensional; CBCT, cone beam computed tomography; kV, kilovoltage; MRI, magnetic resonance imaging; MV, megavoltage.

∗ ‘No action limit’ protocol: first three to five fractions then weekly.

† Percentage may not add to 100% due to rounding.

### Exploratory Questions

Two exploratory questions regarding contouring of bowel structures and image guidance were included within the survey to address the most contentious issues within the working group discussions. All centres, including those not currently using IMRT, were invited to respond to these questions.

Thirty-four of 44 centres (77%) indicated that it would be potentially feasible to implement routine delineation of individual small bowel loops. Despite agreeing that this would be potentially feasible, eight centres indicated that there might be barriers to this implementation in their free text responses and six centres (6/44, 14%) were uncertain whether it would be feasible. Of those six centres, three are not currently using IMRT and two are not currently outlining any bowel structure. Of the four remaining centres (4/44, 9%) that responded that it would not be feasible to implement routine delineation of individual small bowel loops, three currently outline a peritoneal cavity/‘bowel space’ structure. Concerns raised within free text comments relating to proposed implementation of small bowel loop delineation included time/resource constraints and a lack of evidence to support the use of this method over other bowel structures.

Thirty-one of 44 centres (70%) indicated that implementation of daily CBCT image guidance for LCRT would be potentially feasible. Eight centres (8/44, 18%) indicated that they were not sure if implementation of daily CBCT image guidance would be feasible. Of these, five centres are not currently using IMRT, two are using a ‘no action limit’ protocol and one carries out imaging on the first three to five fractions only. Of the remaining five centres (5/44, 11%) that reported it would not be feasible, three are currently using a ‘no action limit’ protocol and one centre is not using IMRT.

### Centres not Currently Using Intensity-modulated Radiotherapy

Eight of 44 centres (18%) indicated that they are not currently using IMRT routinely for the treatment of patients with rectal cancer. Three of these eight centres indicated that they were in the process of implementing IMRT at the time of their survey response, one centre indicated that implementation had been halted due to the COVID-19 pandemic and four centres suggested that there was an insufficient evidence base to prioritise IMRT implementation. Five centres indicated that national guidance would help with future IMRT implementation and all eight responded that guidance would be useful to inform dose fractionation schedules. Recommendations concerning OAR definition, treatment planning and image-guided radiotherapy were also considered to be valuable.

## Discussion

This survey has illustrated the current extent to which IMRT is used in the UK for rectal cancer treatment and the heterogeneity in its implementation and delivery. Interestingly, 81% of centres that responded to the survey are already using IMRT in some form. However, it should be acknowledged that although we consider that the response rate of 70% provides a good overview of practice throughout the country, the results of the survey could still be influenced by response bias, in particular from non-responders who are not currently using IMRT.

This survey represents the first reported comprehensive analysis of IMRT use for rectal cancer in the UK. A previous analysis of the Radiotherapy Dataset and National Cancer Data Repository concerned data from the era before IMRT was widely adopted in the UK [16]. Data describing IMRT use from the National Cancer Registration and Analysis Service (NCRAS) suggest that about 60% of rectal cancer radiotherapy treatments were with IMRT in 2018–2019, which may be comparable with our findings for IMRT use if non-responding centres are taken into consideration [17]. There are limited data concerning IMRT use for rectal cancer internationally. Two analyses of the National Cancer Database and National Comprehensive Cancer Network (NCCN) Colorectal Cancer Database in the USA were recently reported, although these studies examined data from 2005–2015. The authors concluded that although the utilisation of IMRT was increasing in the USA, there remained considerable variability in its uptake. Wegner and colleagues [18] estimated that IMRT use was 22% in the USA in 2014, based on an analysis of the National Cancer Database, and Reyngold *et al.* [19] reported that IMRT treatments at designated NCCN centres represented 38% of all rectal cancer treatments in 2011.

This survey has indicated that there is good consensus among UK centres regarding several aspects of the IMRT treatment pathway, including supine positioning, administration of intravenous contrast and use of bladder filling protocols. All centres reported that they use MRI as an aid in target volume delineation, but few centres use computed tomography/MRI fusion or carry out a radiotherapy-specific MRI.

During the development of the National Rectal Cancer IMRT Guidance, there were several aspects for which it was challenging to secure consensus within the working group due to a lack of existing high-level evidence. In these instances, the survey provided useful information regarding current practice to inform the guidance recommendations. The main issues are outlined below.

(i) Use of a boost and boost dose

The survey indicated that nearly 70% of centres are already using a boost for at least some patients with rectal cancer, with 60% delivering this as a SIB and 36% using doses >50 Gy. Twenty-eight per cent of centres currently use a sequential boost. Taking these survey results into account, and in order to maximise the potential resource benefits of IMRT, including reduced planning time and fewer treatment fractions, the guidance working group have suggested using a SIB rather than a sequential boost. In addition, an SIB may improve patient outcomes as shown in other tumour types [20,21]. The optional boost dose in the guidance is 50 Gy. Given the evidence for a dose–response relationship in rectal cancer, in selected patients or sites of disease where the aim is complete pathological response (e.g. an organ preservation strategy or where pathologically involved nodes are outside of the TME volume), doses >50 Gy can be considered [[21], [22], [23]]. Doses >50 Gy have been examined within several phase II studies, which appear to show acceptable acute toxicities and promising pathological complete response rates, although there remains an absence of phase III data [[24], [25], [26]]. The guidance states that it is at the discretion of individual centres whether they choose to deliver doses exceeding 50 Gy in clinical scenarios where the goal is complete response. We would suggest a SIB of 52 Gy based on a small series showing minimal acute toxicity [21].

(ii) Rectal protocol

Although nearly 60% of centres that responded to the survey indicated that they currently make attempts to minimise rectal filling at simulation, only one third attempt to maintain this during treatment. It has been shown that there is considerable internal organ motion and deformation of the rectum and mesorectum during radiotherapy, which provides the rationale for attempting to standardise rectal volume at simulation and during treatment, especially when a SIB is used during LCRT [27,28]. A suggested rectal protocol is therefore included within the national guidance.

(iii) Target volume definition

All centres currently using IMRT that responded to the survey have a protocol to guide target volume definition, but the references for these protocols varied. The ARISTOTLE trial protocol [8] was cited by 21 centres as a source for target volume delineation guidance. However, the ARISTOTLE trial used a three-dimensional conformal radiotherapy technique, with target outlines encompassing significant volumes of normal tissue [8]. Although not specifically addressed within this survey, it is possible that some centres currently using IMRT, especially those using ARISTOTLE-based protocols, may not be optimising treatments to IMRT-specific target volumes. This may negate the potential benefits of IMRT regarding target dose conformality and reduction in dose to OAR. The guidance provides recommendations for IMRT-specific target volumes (and inverse planning optimisation), which represent a departure from those used in the ARISTOTLE trial. The final volumes included in the guidance were based on a synthesis of clinical trial protocols, consensus guidelines, in-house departmental protocols, a survey of variations in clinical target volumes with rectal cancer staging and a project examining the most reliable method for identifying the superior border of the elective volume during the guidance development [9,12,29]. A comprehensive literature review was undertaken to inform the guidance recommendations for planning target volume margins that account for internal motion and other sources of error and whether daily or a ‘no action limit’ protocol for treatment verification is to be used [30]. With the use of highly conformal target volumes, routine use of quality assurance of radiotherapy contours is of critical importance. Only 39% of centres reported that they are currently using routine peer review of contours, despite recommendations from the Royal College of Radiologists regarding peer review for rectal cancer radiotherapy within a curative intent treatment pathway [31]. As a result, the guidance makes a specific recommendation regarding implementation of routine quality assurance for target volume delineation.

(iv) Small bowel delineation

There was considerable discussion within the working group regarding delineation of individual small bowel loops versus a ‘bowel cavity’ structure. Heterogeneity in practice was also identified in the survey, with several approaches to bowel contouring described, including not delineating a bowel structure at all. This variable approach to bowel contouring reflects a lack of data on the optimal bowel structure to delineate in order to predict and reduce early and late bowel toxicities [15,32,33]. Most centres (77%) considered that delineation of individual bowel loops would be potentially feasible, although we note the comments regarding time and resource implications, as well as limited evidence within the literature as to the optimum approach. Several centres specifically suggested that training radiographers, dosimetrists or physicists to contribute to OAR outlining may be a potential solution to resource constraints. Although there was a strong desire within the working group to recommend a single approach to small bowel delineation, no majority consensus in favour of either delineation of individual bowel loops or ‘bowel cavity’ could be reached. As a compromise, the guidance recommends small bowel loops with dose constraints based on the RTOG 0822 trial [12]. However, alternative delineation instructions and constraints for a ‘bowel cavity’ structure are also included within an Appendix in the National Rectal Cancer IMRT Guidance.

(v) Organ at risk dose constraints

Few IMRT-specific OAR constraints are reported within the literature for rectal cancer, especially for SCRT delivering 25 Gy in five fractions. This is particularly relevant because during the current COVID-19 pandemic there has been an increased use of SCRT (with or without sequential neoadjuvant chemotherapy) as an alternative to LCRT [34]. The survey indicated that centres are currently using a variety of sources for OAR constraints, including the National Anal Cancer IMRT Guidance [14] and anal cancer clinical trial protocols as well as the ARISTOTLE protocol [8] (which did not actually contain OAR constraints) [8,9,[12], [13], [14], [15]]. The working group noted that OAR constraints from the National Anal Cancer IMRT Guidance were already in general use and therefore decided to use these as a basis for many of the guidance recommendations [14]. For SCRT, no robust recommendations could be found in the literature; instead, in-house constraints from two UK centres were included in the guidance, as these were known to work in clinical practice.

(vi) Treatment verification

Although 70% of centres considered that implementation of daily CBCT would potentially be feasible, several respondents highlighted the potential resource implications of such a recommendation. The guidance therefore mandates daily online CBCT for SCRT and recommends daily online imaging with CBCT for LCRT delivered with IMRT. Acknowledging that this recommendation may not be feasible in all centres, two sets of recommended planning target volume margins for LCRT are provided for a daily and a ‘no action limit’ protocol, respectively.

Heterogeneity in the use of radiotherapy for rectal cancer treatment during the pre-IMRT era was demonstrated in an anslysis of the Radiotherapy Dataset and National Cancer Data Repository[16]. This study showed there was variation between NHS Trusts in England regarding whether radiotherapy was used, what type of radiotherapy was delivered (short course versus long course) and the time interval between radiation and surgery. The authors suggested that this variation in clinical decision making between multidisciplinary teams across England might be explained by differing levels of confidence and expertise among surgeons and radiologists. Since then, the evidence base for the optimal use of radiotherapy has continued to develop [16,35] and in recent years, a radiotherapy modernisation programme has taken place across the UK. This programme has encouraged the adoption of IMRT, but there has not been clear national guidance on its optimal implementation for rectal cancer [36,37]. It is possible that an absence of national guidance may account for some of the variation in practice demonstrated in our survey. This ongoing heterogeneity in IMRT use provides a rationale for attempting to harmonise practice by implementing national guidance. It will be important to evaluate the impact of the guidance on patient outcomes, for example using NCRAS data or national audits such as those carried out following publication of the National Anal Cancer IMRT Guidance [17,[38], [39], [40]]. These types of evaluations would allow comparison of clinical practice alongside clinicopathological and outcome data and provide the clearest understanding of whether practice has changed and the impact this has had on patient care.

In summary, the survey results reported in this paper have provided valuable insight into the current landscape of IMRT use for rectal cancer in the UK and have helped frame the recommendations contained in the first version of the National Rectal Cancer IMRT Guidance [7]. We hope that the National Rectal Cancer IMRT Guidance will encourage implementation of IMRT in all centres across the UK and help to harmonise the variation in practice identified by the survey. We plan to undertake a further survey following release of the guidance to evaluate the impact of its recommendations and to inform development of further versions of the guidance.

## Conflicts of Interest

The authors declare no conflicts of interest.

## Funding

This research did not receive any specific grant from funding agencies in the public, commercial, or not-for-profit sectors.
