# Supplementary material for: Intensity-modulated Radiotherapy for Rectal Cancer in the UK in 2020
Source: Clin Oncol (R Coll Radiol). 2021 Apr;33(4):214–23. doi: 10.1016/j.clon.2020.12.011 (PMC7985673; doi:10.1016/j.clon.2020.12.011)
Supplement: Multimedia component 1 [file mmc1.docx]

**Table S1**

A comparison of treatment technique, target volume delineation and organ at risk constraints between the ARISTOTLE clinical trial and the new National Rectal Cancer IMRT Guidance

|  | *ARISTOTLE* | *National Rectal Cancer IMRT Guidance* |
| --- | --- | --- |
| *Technique* | 3D CRT | IMRT |
| *GTV primary* | Whole rectum included at involved level | Tumour alone included (if clearly identifiable) |
| *CTV* | CTV A=GTV +10 mm  CTV B=mesorectum (+ 10 mm anterior margin), presacral, internal iliac and obturator nodal volumes  *Note*: CTV B has a flat anterior border. Border of internal iliac nodes determined by 7 mm margin around vessels  CTV F= CTV A + CTV B | ICTVp= GTV primary + 10 mm  ICTVn= GTV nodes + 5 mm  ICTV_Elec= mesorectum (+ 10 mm anterior margin), presacral, internal iliac, obturator and inferior meseneteric/superior rectal artery nodal volumes  *Note:* ICTV_Elec is a highly conformal volume. Anterior border of presacrum nodes determined by 10 mm margin anterior to sacrum; border of internal iliac nodes determined by 7 mm margin around vessels; medial border of obturator nodes determined by 17 mm margin medial to obturator internus muscle  ICTV_Final= ICTVp + ICTVn + ICTV_Elec |
| *Superior border of CTV* | Level of S2/3 vertebral interspace (or 2cm superior to the GTV) | Presacrum: level of S1/2 vertebral interspace (or 2 cm superior to the highest involved node)  Mesorectum: level of S2/3 vertebral interspace or, where identifiable, bifurcation of superior mesenteric artery into inferior mesenteric artery and sigmoid artery |
| *PTV* | CTV F + 10 mm | ICTV_Final + 5 mm (daily online volumetric imaging verification)  ICTV_Final + 10 mm (offline imaging verification) |
| *OAR constraints* | No specific OAR constraints | IMRT specific OAR constraints included for bladder, bowel cavity/bowel loops and femoral heads |

3D CRT, 3 dimensional conformal radiotherapy; CTV, clinical target volume; GTV, gross tumour volume; ICTV_Elec, internal clinical target volume elective; ICTVn, internal CTV nodes; ICTVp, internal CTV primary; IMRT, intensity modulated radiotherapy; PTV, planning target volume
