# Supplementary material for: Intensity-modulated Radiotherapy for Rectal Cancer in the UK in 2020
Source: Clin Oncol (R Coll Radiol). 2021 Apr;33(4):214–23. doi: 10.1016/j.clon.2020.12.011 (PMC7985673; doi:10.1016/j.clon.2020.12.011)
Supplement: Multimedia component 2 [file mmc2.pdf]

Clinical Oncology

The Royal College of Radiologists

# Rectal IMRT Survey 2020

## Introduction

The aim of this survey is to establish the current use of advanced radiotherapy techniques in rectal cancer and to support excellent, safe patient care by using the results of this survey to inform the development of a national intensity-modulated radiation therapy (IMRT) guideline for rectal cancer. (NB: If your centre does not currently use IMRT to treat patients with rectal cancer, you can still participate: you will only need to complete part of the survey.)

We would like to receive one response per centre and we would recommend you work with other members of the team where necessary. We anticipate that some sections may require input from clinical oncologist, physicist/planning and radiographer and we have indicated suggested team members for each section.

The closing date for receipt of survey responses is **Monday 6 April**. Depending on your answers, the survey should take a maximum of 10 minutes to complete.

This survey will ask you questions regarding the following:

1. Radiotherapy technique
2. IMRT dose prescription
3. IMRT pre-treatment activities
4. IMRT target volume and organs at risk delineation (and treatment planning)
5. IMRT treatment verification.

All questions concern patients treated using IMRT/volumetric modulated arc therapy (VMAT), even if this is a minority of your patients. For those patients, we want to know how you treat <u>routine</u> cases, and not unusual or exceptional cases.

If you have any questions about this survey, please contact the Projects Co-ordinator, Audit & Surveys - audit_QI@rcr.ac.uk / 020 7406 5956. For clinical questions, please contact Dr Rebecca Muirhead, rebecca.muirhead@oncology.ox.ac.uk.

Clinical Oncology
The Royal College of Radiologists

# Rectal IMRT Survey 2020

## Centre/Hospital ID and Name

* 1. Please select your centre/hospital ID number (provided by the RCR):

* 2. Please select your centre/hospital name:

* 3. Please enter your email address:

Clinical Oncology
The Royal College of Radiologists

# Rectal IMRT Survey 2020

## Section 1: Radiotherapy Technique
(clinical oncologist, physicist/planning, radiographer)

* 4. Does your centre/hospital use IMRT/VMAT (including either static beam or arc therapy) to treat patients with rectal cancer?

- ◯ Yes GO TO Q8
- ◯ No

# Rectal IMRT Survey 2020

* 5. What are the primary barriers to your centre/hospital implementing IMRT? (please tick all that apply, and provide further details where possible)

- ☐ Delivery pathway (e.g. image-guided radiation therapy (IGRT) limitations or insufficient capacity to deliver IMRT)
- ☐ Outlining pathway (e.g. clinician time, staff availability or contouring training)
- ☐ Planning pathway (e.g. IMRT licences or physicist/dosimetrist staff availability or training)
- ☐ Treatment QA resources
- ☐ Insufficient evidence to prioritise implementation of IMRT
- ☐ Insufficient funding to implement and deliver IMRT
- ☐ No local leadership to coordinate implementation
- ☐ Other (please specify) / further details:

* 6. Would a UK-specific IMRT guidance document help your centre/hospital implement IMRT?

- ○ Yes
- ○ No
- ○ Not sure

If No/Not sure, please provide further details:

Clinical Oncology

The Royal College of Radiologists

# Rectal IMRT Survey 2020

*** 7. How might a UK-specific IMRT guidance document help your centre/hospital develop/improve your current IMRT protocol?**
(please tick all that apply)

- [ ] Image-guided radiotherapy
- [ ] IMRT prescription including use of a boost volume
- [ ] Organ at risk (OAR) definition
- [ ] Pre-treatment activities including bladder/rectal filling
- [ ] Target volume definition
- [ ] Treatment planning
- [ ] Other (please specify):

**GO TO Q35**

Clinical Oncology

The Royal College of Radiologists

# Rectal IMRT Survey 2020

* 8. Do you treat all of your rectal cancer patients with IMRT/VMAT?

- ◯ Yes
- ◯ No, most patients are treated with three-dimensional conformal radiation therapy (3D-CRT), but patients with potentially large OAR exposure receive IMRT/VMAT
- ◯ No, most patients are treated with 3D-CRT, but patients receiving a boost get IMRT/VMAT
- ◯ No, most patients are treated with 3D-CRT, but some patients get IMRT/VMAT (please provide details below)
- ◯ No, most patients are treated with IMRT/VMAT, but some patients receive 3D-CRT (please provide details below)
- ◯ Other (please specify) / additional details:

Clinical Oncology
The Royal College of Radiologists

# Rectal IMRT Survey 2020

## Section 2: IMRT Prescription Details
(clinical oncologist, physicist/planning)

* 9. For long-course chemoradiotherapy (LCRT), which of the following dose prescriptions does your centre/hospital you use for the elective treatment volumes?
(please tick all that apply)

- [ ] 45 Gy in 25 fractions
- [ ] 50 Gy in 25 fractions
- [ ] 50.4 Gy in 28 fractions
- [ ] Other dose/fractionation (please specify):

10. If you indicated more than one dose prescription above, which is the most common?

* 11. For LCRT, does your centre/hospital routinely deliver a boost to a restricted target volume?

- ○ Yes, for all or nearly all patients
- ○ Yes, but only for selected patients
- ○ No

# Rectal IMRT Survey 2020

* 12. What total dose will be delivered to the boost volume?

* 13. What total number of fractions will be delivered to the boost volume?

Clinical Oncology
The Royal College of Radiologists

# Rectal IMRT Survey 2020

## Section 3: IMRT Pre-Treatment Activities
(clinical oncologist, radiographer)

* 14. What is the standard treatment position in your centre/hospital, as defined in your IMRT protocol?

- ◯ Prone
- ◯ Supine

* 15. Does your centre/hospital have a bladder protocol?

- ◯ Yes, patients are simulated and treated with empty bladder
- ◯ Yes, patients are asked to drink 250-600 ml of water 20-40 minutes prior to simulation and treatment
- ◯ Yes, other bladder filling protocol used
- ◯ No

Clinical Oncology
The Royal College of Radiologists

# Rectal IMRT Survey 2020

**16. Please provide additional details regarding how bladder filling is monitored and adapted during treatment.**

Clinical Oncology

The Royal College of Radiologists

## Rectal IMRT Survey 2020

*** 17. Does your centre/hospital have a rectal protocol for simulation?**

- ◯ Yes, patients are asked to empty their bowels prior to simulation
- ◯ Yes, patients are given micro-enema prior to simulation
- ◯ Yes, rectal filling (gas/air) is assessed on the CT scout view, and intervention taken if necessary
- ◯ Yes, other rectal filling protocol used for simulation
- ◯ No

Clinical Oncology
The Royal College of Radiologists

# Rectal IMRT Survey 2020

**18. Please provide further details of your rectal protocol for simulation.**

Clinical Oncology

The Royal College of Radiologists

# Rectal IMRT Survey 2020

* 19. Does your centre/hospital attempt to maintain your rectal filling protocol throughout treatment?

- ◯ Yes
- ◯ No

Clinical Oncology

The Royal College of Radiologists

# Rectal IMRT Survey 2020

**20. Please provide additional details regarding how rectal filling is monitored and adapted during treatment.**

Clinical Oncology
The Royal College of Radiologists

# Rectal IMRT Survey 2020

*** 21. For which patients does your centre/hospital use intravenous contrast?**

- ◯ All patients (unless contraindicated)
- ◯ Only selected patients
- ◯ None

Clinical Oncology
The Royal College of Radiologists

# Rectal IMRT Survey 2020

**22. Please provide details of which patients your centre/hospital uses intravenous contrast for.**

Clinical Oncology

The Royal College of Radiologists

# Rectal IMRT Survey 2020

* 23. For which IMRT patients does your centre/hospital use oral small bowel contrast?

- ◯ All patients (unless contraindicated)
- ◯ Only selected patients
- ◯ None

# Rectal IMRT Survey 2020

**24. Please provide details of which patients your centre/hospital uses oral small bowel contrast for.**

# Rectal IMRT Survey 2020

## Section 4: IMRT Target Volume Definition and Planning
(clinical oncologist, physicist/planning)

* 25. Which protocol, if any, does your centre/hospital base its target volume outlining on?
(please tick all that apply: if using a local protocol primarily based on any of the guidelines/protocols listed below, please tick BOTH the guideline/protocol box AND the locally-developed protocol box)

- [ ] ARISTOTLE trial guideline
- [ ] Roels et al (IJROBP 2006)
- [ ] Valentini et al (Radiother Oncol 2016)
- [ ] Other trial or published guideline (please provide details below)
- [ ] Locally developed protocol
- [ ] No outlining protocol
- [ ] Other (please specify) / further details:

*** 26. Does your centre/hospital routinely use MRI to guide target volume delineation?**

- ◯ Yes, diagnostic magnetic resonance imaging (MRI) (co-registered to planning computed tomography (CT))
- ◯ Yes, diagnostic MRI (not co-registered to planning CT, used side-by-side)
- ◯ Yes, radiotherapy specific MRI (co-registered to planning CT)
- ◯ Yes, radiotherapy specific MRI (not co-registered to planning CT, used side-by-side)
- ◯ Yes, MRI-only planning pathway
- ◯ No
- ◯ Other (please specify):

*** 27. Does your centre/hospital have routine peer review of contours?**

- ◯ Yes
- ◯ No

*** 28. Which of the following organs at risk does your centre/hospital routinely outline?**
(please tick all that apply)

- [ ] Bladder
- [ ] Bowel/peritoneal cavity (bowel bag)
- [ ] Bowel, other structure (please provide details below)
- [ ] External genitalia (e.g. scrotum, penile shaft, clitoris, labia) (please provide details below)
- [ ] Femoral heads
- [ ] Individual large bowel loops
- [ ] Individual small bowel loops
- [ ] Internal genitalia (e.g. vagina, penile bulb)
- [ ] Sigmoid
- [ ] Other (please specify) / further details:

Clinical Oncology
The Royal College of Radiologists

# Rectal IMRT Survey 2020

## Section 5: IMRT Treatment Planning
(clinical oncologist, physicist/planning)

*** 29. Does your centre/hospital use OAR constraints/objectives for plan optimisation and/or assessment?**

- ◯ Yes, OAR constraints based on clinical trial protocol used (please provide name of trial below)
- ◯ Yes, in-house OAR constraints used (please provide details below)
- ◯ No, but we still try to minimise dose to OARs
- ◯ No

Other (please specify) / name of trial / further details:

*** 30. Does your centre/hospital utilise a static IMRT or VMAT technique for treatment delivery?**

- ◯ Static IMRT
- ◯ VMAT
- ◯ Other (please specify):

Clinical Oncology
The Royal College of Radiologists

# Rectal IMRT Survey 2020

## Section 6: IMRT Treatment Verification
(clinical oncologist, radiographer)

* 31. Which imaging modalities does your centre/hospital use for on-treatment verification?
(please tick all that apply)

- [ ] 2D kV imaging
- [ ] Combined kV-MV 2D imaging
- [ ] Cone beam CT
- [ ] MRI
- [ ] On board CT
- [ ] Portal 2D MV imaging
- [ ] Other (please specify):

32. If you use a combination of on-treatment imaging modalities, please provide details of how they are combined.

* 33. Which on-treatment imaging schedule does your centre/hospital routinely use for LCRT patients?

◯ First treatment fraction only

◯ First 3-5 treatment fractions

◯ First treatment fraction, and weekly

◯ First 3-5 treatment fractions, and weekly (‘no action level protocol’)

◯ Daily imaging

◯ Don't know

◯ Other (please specify):

* 34. Did your centre/hospital's on-treatment IGRT verification protocol change when you transitioned from 3D-CRT to IMRT/VMAT?

◯ Yes

◯ No

If yes, please specify:

Clinical Oncology

The Royal College of Radiologists

# Rectal IMRT Survey 2020

35. Regarding organs at risk outlining, we are reviewing the available evidence and discussing whether to recommend delineation of the peritoneal cavity or individual small/large bowel loops. These discussions have focused on the additional time potentially involved in delineation of individual loops considering the conflicting evidence available on the different approaches to contouring bowel and toxicity in the literature.

If delineation of individual bowel loops was to be recommended in a national IMRT rectal cancer guideline, would it be feasible for you to undertake this for every routine case within your centre/hospital?

- Yes
- No
- Don't know

Additional comments:

**36. We are discussing whether to recommend daily online imaging for all IMRT rectal cancer treatments, taking into account the greater conformality of target volumes used for IMRT in comparison to 3D-CRT.**

**If daily image verification using cone beam CT was to recommended for all IMRT rectal cancer treatments, would it be feasible for you to undertake this for every routine case within your centre/hospital?**

- ◯ Yes
- ◯ No
- ◯ Don't know

Additional comments:

# Rectal IMRT Survey 2020

**37. Please provide further clarification for any of your responses, or give any further comments here.**

Clinical Oncology

The Royal College of Radiologists

# Rectal IMRT Survey 2020

Thank you for participating in this survey: please now click **Submit** to send us your responses.

Your responses will illustrate how IMRT is used in rectal cancer across the UK, and this will help inform our development of a national IMRT guideline for rectal cancer.
